# A Novel Multiaxial Strain-Based Criterion Considering Additional Cyclic Hardening

**DOI:** 10.3390/ma14102542

**Published:** 2021-05-13

**Authors:** Sabrina Vantadori

**Affiliations:** Department of Engineering & Architecture, University of Parma, Parco Area delle Scienze 181/A, 43124 Parma, Italy; sabrina.vantadori@unipr.it; Tel.: +39-05-2190-5962

**Keywords:** additional cyclic hardening, critical plane, LCF, Ti-6Al-4V, non-proportional loading

## Abstract

The present paper is dedicated to the theoretical evaluation of a loading feature, that may have a significant influence on fatigue phenomenon: non-proportionality. As a matter of fact, considerable interactions between dislocations, leading to the formation of dislocation cells, cause additional cyclic hardening of material. Such a phenomenon is experimentally observed for materials sensitive to non-proportionality. In such a context, the present paper is aimed to propose a novel multiaxial strain-based criterion, the refined equivalent deformation (RED) criterion, which allows to take into account, in fatigue life estimation, both strain amplitude and additional cyclic hardening. The accuracy of the novel criterion is evaluated by considering experimental tests, performed on Ti-6Al-4V specimens, subjected to multiaxial LCF loading.

## 1. Introduction

It is well known that one feature of loading that may have a significant influence on fatigue phenomenon is the non-proportionality of a load [1], where a rotation of principal stress or strain axes occurs during the observation time interval. As a matter of fact, under the influence of non-proportional loading, shear stresses, acting in multiple directions and planes, activate additional slip systems in uniaxial or multiaxial proportional loading. Therefore, new sources of dislocation appear. Considerable interactions between dislocations (that do not appear under proportional loading) result in a high density, leading to the formation of dislocation cells, causing additional cyclic hardening of materials, which usually has a significant negative effect on the lifetime.

As a result, a load characterized by a high degree of non-proportionality, acting on a material with a high susceptibility to non-proportionality, can lead to as much as a 10-fold decrease in fatigue strength with respect to proportional loading [1].

Therefore, the theoretical evaluation of the effect of the strain path non-proportionality on the ultimate state of a material under fatigue loading represents an interesting research field for engineering practice, and, consequently, at a stage of dynamic development.

It is worth noticing that a fatigue criterion cannot accurately estimate fatigue life by only considering the stress/strain state inside a material in terms of the computation of a suitable damage parameter. Among the criteria available in the literature for such an evaluation, both strain- and energy-based criteria are widely used [2,3,4,5,6,7,8,9,10,11,12,13,14,15,16,17].

In such a context, the present paper is aimed to propose a novel multiaxial strain-based criterion, named Refined Equivalent Deformation (RED) criterion, that allows to take into account in fatigue life estimation both the strain amplitude and additional cyclic hardening when the load is non-proportional and the material is sensitive to non-proportionality. More precisely, an enhancement factor is implemented in the damage parameter relationship, proposed in [18]. Such a factor is a function of material constants, strain path orientation, and the degree of non-proportionality [3,4,5].

The accuracy of the proposed criterion is verified by considering an experimental campaign, available in the literature [6], performed on a material that is sensitive to loading non-proportionality and is widely used in industry: titanium alloy. As a matter of fact, titanium alloys are widely used in aerospace, automotive, and biomedical and chemical industries due to their superior properties, such as their corrosion resistance and high strength-to-weight ratio [19,20,21,22,23]. In such a context, Ti-6Al-4V here examined has excellent tensile (tensile strength ≥ 895 MPa) and fatigue strength (equal to about 460MPa at a number of loading cycles of 109 under rotating bending), as well as high resistance to a wide spectrum of corrosive environments, with respect to other titanium based alloys, due to its inclination to form protective surface oxides [24,25,26,27,28,29,30,31,32,33,34,35]. As such, it is used in life-limit components of civil aviation engines and as fractured critical components of military engines [36,37,38,39]. Therefore, fatigue behavior, especially under low-cycle fatigue (LCF) regimes, should be critically evaluated for the design of the above-mentioned components under service loads. More precisely, such components are subjected to cyclic loading due to thermal and mechanical stresses, which cause multiaxial LCF that may be proportional and/or non-proportional [40,41,42].

A comparison between the experimental data and the theoretical results, in terms of fatigue lifetime, is performed, and the influence of the phase shift on fatigue strength is investigated. A comparison with the results obtained by applying other criteria available in the literature is also carried out.

## 2. Examined Experimental Campaign

The examined experimental campaign [6] is presented. More precisely, uniaxial and multiaxial fatigue tests under LCF regime were performed on TC4 titanium alloy specimens.

### 2.1. Material and Specimens

The material was TC4 titanium alloy, and was produced in China [6]. A similar material produced in the USA is Ti-6Al-4V.

The chemical composition is reported in Table 1, and its mechanical properties are: elastic modulus E=108.4 GPa, Poisson’s ratio $\nu_{e}=0.25$, and yield stress $\sigma_{y}=942.5\;MPa$ [6].

**Figure 1:**
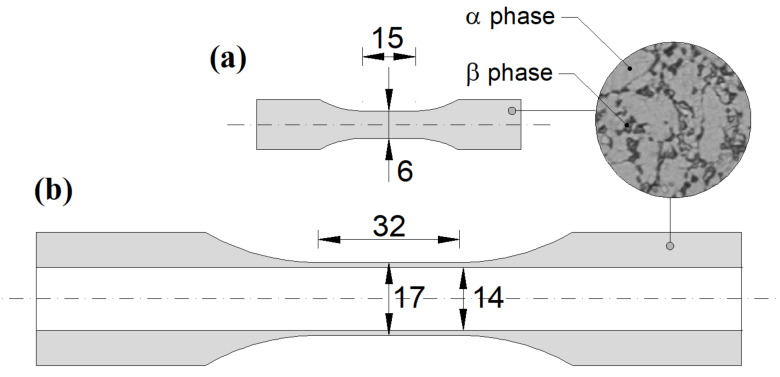
Specimens employed for (**a**) axial fatigue tests and (**b**) torsional and combined axial/torsional tests. The material microstructure is also reported: alpha grains (light) with intergranular beta phase (dark).

Heat treatment, which was performed on the material before machining, consisted in heating to 730 °C and then air cooling [6]. After such a treatment, the material microstructure (Figure 1) was homogeneous and consisted of fully equiaxed and columnar alpha grains (light) with intergranular beta phase (dark).

The specimens were machined from full bars with a diameter of 35 mm [6]. Two types of specimen were produced: solid specimens with a diameter equal to 6 mm along a gauge length of 15 mm (Figure 1a), employed for axial fatigue tests (according to ASTM standard ASTM E606/E606M), and hollow specimens with an outer diameter equal to 17 mm and an inner diameter equal to 14 mm, both along a gauge length of 32 mm (Figure 1b), employed for torsional and combined axial/torsional tests (according to ASTM standard E2207).

### 2.2. Testing Conditions

LCF tests were performed using a servo-hydraulic MTS Model 809 testing system [6]. The tests were carried out under the strain-controlled mode of loading, where an axial-torsional extensometer was used to measure the axial and shear strains. Axial load and torque were also registered during testing.

Axial, torsional, and combined axial–torsional fatigue tests were characterized by a loading ratio equal to −1, and sinusoidal waveforms with a frequency between 0.5 and 1.0 Hz. Under multiaxial loading conditions, both proportional and non-proportional signals were investigated, the latter was characterized by a phase shift, β, equal to 45° or 90°.

The failure criterion was considered when a 10–15% drop in axial load or torque was observed (whichever occurred first) with respect to the corresponding values registered at midlife.

### 2.3. Experimental Results

**Figure 2:**
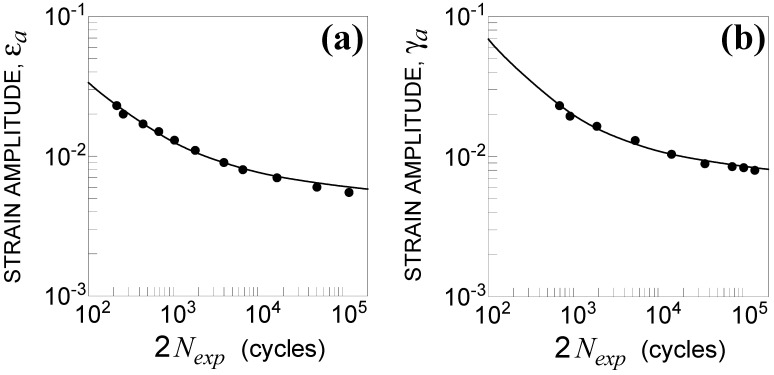
Uniaxial experimental results and Manson–Coffin curves under (**a**) tension and (**b**) torsion.

The experimental results under uniaxial loading are shown in Figure 2, whereas those under multiaxial loading are listed in Table 2, where $\varepsilon_{a}$ is the measured axial strain amplitude, $\gamma_{a}$ is the measured shear strain amplitude, β is the phase shift, and $N_{\exp}$ is the fatigue life.

It was observed that the fatigue life under both axial and torsional loading was well correlated by the Manson–Coffin equations [6], in which the parameters were ${\sigma^{\prime }}_{f}=1116.9\;MPa$, b=−0.049, ${\varepsilon^{\prime }}_{f}=0.58$, c=−0.679 and ${\tau^{\prime }}_{f}=716.9\;MPa$, $b_{0}=-0.060$, ${\gamma^{\prime }}_{f}=2.24$, and $c_{0}=-0.800$, respectively.

## 3. Simulation of the Experimental Tests

It is well known that changing of principal axes under non-proportional loading can lead to additional cyclic hardening, which is considered to be closely related to decreases in the fatigue life. Such a phenomenon is more pronounced when a material is sensitive to loading non-proportionality.

A fatigue criterion cannot estimate fatigue life by only considering the stress/strain state in computations of damage parameters because, under non-proportional loading, the rotation of principal stress or strain axes occurs and new sources of dislocation appear. Considerable interactions between dislocations lead to the formation of dislocation cells, causing additional cyclic hardening of material, which usually has a significant negative effect on the lifetime.

In such a context, an enhancement factor, implemented in the damage parameter relationship, can be used to reflect the effect of additional cyclic hardening on the estimated fatigue life.

The reduced strain range method proposed by Borodii et al. [3,4,5] is a strain-based criterion that, uses an enhancement factor (named strain factor in the following), which is the function of the material constants, strain path orientation, and degree of non-proportionality. In Section 3.1, such a method is presented and applied to the experimental campaign examined here [6], highlighting two drawbacks of the method.

The novelty of the present paper is to propose a strain-based criterion, the RED criterion, which is feasible for estimation of the multiaxial life of metallic engineering components, especially under non-proportional loading. More precisely, as presented in Section 3.2, a strain factor, along the lines of that proposed by Borodii et al., but which overcomes the above-mentioned drawbacks [3,4,5], is implemented in the damage parameter relationship proposed by Vantadori et al. [18].

### 3.1. The Reduced Strain Range Method

According to the method proposed by Borodii et al. [3,4,5], experimental fatigue data under non-proportional loading can be represented using the reduced strain range, $\Delta \varepsilon_{np,i}$, which for the *i*-th test is given by:

(1) $$\Delta \varepsilon_{np,i}=f_{i}\;\Delta \varepsilon_{ASME,i}$$

where $f_{i}$ is the strain factor and $\;\Delta \varepsilon_{ASME,i}$ is the standard definition of the strain range [43], computed as:

(2) $$\Delta \varepsilon_{ASME,i}=max\left[\sqrt{{\left(\varepsilon_{A,i}-\varepsilon_{B,i}\right)}^{2}+\frac{1}{3}{\left(\gamma_{A,i}-\gamma_{B,i}\right)}^{2}}\right]$$

where $\varepsilon_{A,i},\varepsilon_{B,i},\gamma_{A,i}$ and $\gamma_{B,i}$ are the total axial and shear strains related to the *i*-th test, at time instants $t_{A}$ and $t_{B}$.

The strain factor, $f_{i}$, is given by:

(3) $$f_{i}=(1+k\;sen\varphi_{i})\;(1+\alpha \;\Phi_{i})$$

where k is the material constant characterizing the difference in the cyclic properties with respect to the proportional strain path, $\varphi_{i}$ is the angle of the *i*-th non-proportional strain path with respect to the material principal axis, α is the material constant related to additional cyclic hardening, and $\Phi_{i}$ is the coefficient of non-proportionality of the *i*-th non-proportional strain path. Note that the material principal axis corresponds to the direction of the proportional strain path where the maximum value of $\Delta \varepsilon_{ASME,i}$ is realized and the longest lifetime is attained. Further details are given in References [3,4,5].

More precisely, the first term in the brackets of Equation (3) takes into account the experimental evidence that when the fatigue life under an uniaxial strain path is greater than that under proportional strain path, a similar trend holds true for non-proportional strain path with respect to the proportional one rotated by the same angle. The material constant, k, is computed by considering experimental data related to tests under proportional loading, uniaxial and/or multiaxial, depending on the material principal axis.

The second term in the brackets of Equation (3) takes into account the experimental evidence that under non-proportional loading the decreasing in fatigue lifetime depends on both the strain range and the cyclic path shape, by means of the coefficient $\Phi_{i}$. Details on the calculation of such parameters are given in References [3,4,5].

Under proportional loading (uniaxial or multiaxial), $\Delta \varepsilon_{np,i}=\Delta \varepsilon_{ASME,i}$. As a matter of fact, in such a case, k is assumed to be equal to zero, according to the corresponding definition given above, whereas $\Phi_{i}$ is equal to zero, being a proportional strain path non-convex.

The constant k is computed through the following expression:

(4) $$k=\sum_{i=1}^{M}\frac{1}{sen\varphi_{i}}\left[{\left(\frac{N_{exp,i}}{N_{f,i}}\right)}^{n}-1\right]$$

where *M* is the tests number under proportional loading, excluding those characterized by a strain path along the material principal direction, whereas the constant α is given by:

(5) $$\alpha =\sum_{i=1}^{N}\frac{1}{\Phi_{i}}\left[{\left(\frac{N_{exp,i}}{N_{f,i}}\right)}^{n}-1\right]$$

where *N* is the number of tests under non-proportional loading.

The fatigue lifetime, $N_{f,i}$, is computed as:

(6) $$N_{f,i}={\left(\frac{f_{i}\;\Delta \varepsilon_{ASME,i}}{10^{B}}\right)}^{1/n}$$

where n and B are the linear approximation coefficients of the experimental data relative to the material principal axis.

#### 3.1.1. Theoretical Results

For the experimental campaign examined here, the values of $\varphi_{i}$, $\Phi_{i}$, and $\Delta \varepsilon_{ASME,i}$ are listed in Table 2. The computed values of the linear approximation coefficients n and B were −0.226 and −0.877, respectively. Therefore, by exploiting the $\varphi_{i}$, $\Phi_{i}$, $N_{exp,i}$ and $\Delta \varepsilon_{ASME,i}$ values, and the n and B coefficients, the material constants k and α were calculated, that is: k=0.9963 and α=0.3214. Note that, to compute k and α, $N_{f,i}$ was calculated according to Equation (6) by assuming $f_{i}=1.0$.

**Figure 3:**
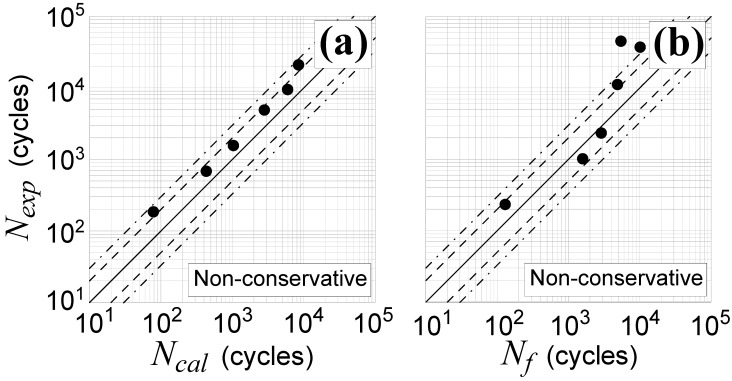
TC4 titanium alloy experimental and estimated lifetime obtained by the reduced strain range method for non-proportional tests with a phase shift, β, equal to: (**a**) 45° (Nos. T7–T12, in Table 2) and (**b**) 90° (Nos. T13–T18, in Table 2).

In Figure 3, the estimated fatigue life, $N_{f}$, which was computed by employing the reduced strain range method, is compared with the experimental one, $N_{exp}$, for non-proportional loading characterized by a phase shift, β, equal to 45° (Figure 3a) or 90° (Figure 3b). Although the results can be considered as quite satisfactory, falling most of the data in scatter band 3, some drawbacks of the method need to be highlighted, as presented in Section 3.1.2.

#### 3.1.2. Criterion Drawbacks

As can be observed in Table 2, a significant additional cyclic hardening is experimentally observed under non-proportional loading: for example, by considering test No. T1 characterized by $\Delta \varepsilon_{ASME}$ = 1.018 under proportional loading, a decreasing in fatigue life of about 55% was registered under a phase shift of β=45° (see test No. T7 with $\Delta \varepsilon_{ASME}$ = 0.996), whereas a decrease of about 76% was registered under a phase shift of β=90° (see test No. T15 with $\Delta \varepsilon_{ASME}$ = 0.998). The same trend was observed by comparing tests No. T3 and T10 and tests No. T9 and T17.

Such experimental evidence was captured by the method proposed by Borodii et al. As a matter of fact, considering test simulation No. T1, with an estimated fatigue life of 84,402 loading cycles (see Table 2), a decrease in fatigue life of about 90% was registered under a phase shift of β=45° (see test simulation No. T7), whereas a decrease of about 94% was registered under a phase shift of β=90° (see test simulation No. T15). The same trend was observed for test simulation No. T10 with respect to No. T3, and for test simulation No. T9 with respect to No. T17.

The drawbacks of the method are related to the results obtained when the loading was the same, but the phase shift was varied from 0° to 90°. Over 90°, the trend is mirrored, and thus theoretical investigations can be avoided.

**Figure 4:**
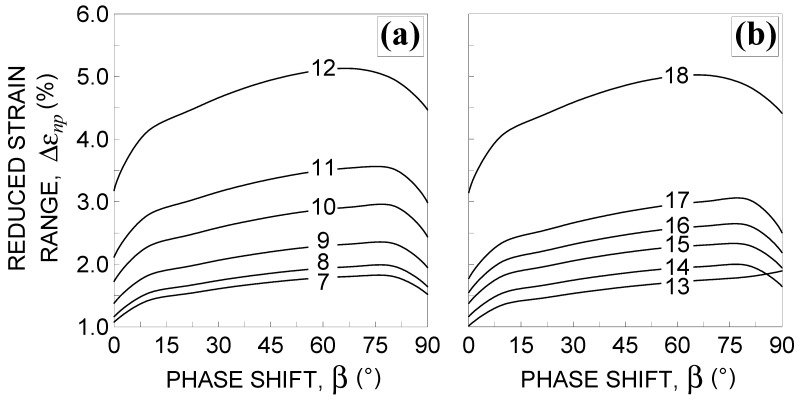
Influence of the phase shift $\beta \;\left(0{}^{\circ}\leq \beta \leq 90{}^{\circ}\right)$ on the reduced strain range $\Delta \varepsilon_{np}$ [3,4,5] for the loading conditions: (**a**) Nos. 7–12 and (**b**) Nos. 13–18, in Table 2.

In Figure 4, the reduced strain range is plotted against the phase shift for the loading conditions from No. 7–No.18, listed in Table 2. It can be observed that $\Delta \varepsilon_{np}$ increases by increasing β to a value of about 80°, and then decreases with the exception of loading condition No. 13.

**Figure 5:**
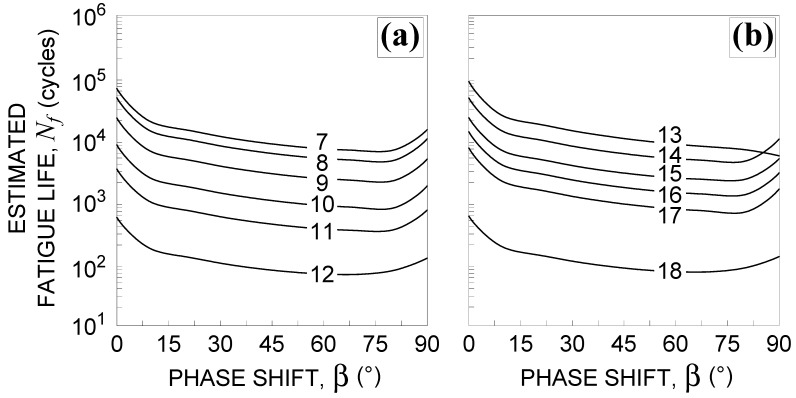
Influence of the phase shift $\beta \;\left(0{}^{\circ}\leq \beta \leq 90{}^{\circ}\right)$ on fatigue life estimation using the Borodii et al. method [3,4,5] for the loading conditions: (**a**) Nos. 7–12 and (**b**) Nos. 13–18, in Table 2.

Consequently, by computing the estimated fatigue life, $N_{f}$, it decreases to the above-mentioned value of β, and then increases with the exception of loading condition No. 13, as shown in Figure 5.

Such a trend does not capture the experimental evidence that recognizes 90° out-of-phase loading as the most damaging loading path.

**Figure 6:**
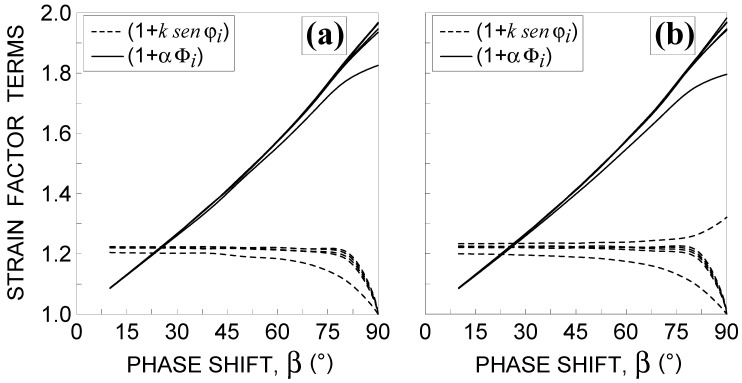
Influence of the phase shift $\beta \;\left(10{}^{\circ}\leq \beta \leq 90{}^{\circ}\right)$ on both terms of the strain factor $f_{i}$ proposed by Borodii et al. [3,4,5] for the non-proportional loading conditions: (**a**) Nos. 7–12 and (**b**) Nos. 13–18, in Table 2.

The second drawback of the criterion is related to the first term in the brackets of Equation (3). By plotting such a term against β for all the examined non-proportional loading conditions (see Figure 6), it can be observed that the curves decrease by increasing the phase shift (with the exception of that corresponding to loading condition No. 13), whereas the opposite occurs for the second terms in the brackets of Equation (3) (also reported in Figure 6).

In order to capture the experimental evidence that the estimated fatigue life has to decrease by increasing the phase shift, the strain factor has to monotonically increase by increasing the phase shift, and such a trend may always be assured (independent of the loading conditions) when both terms in Equation (3) increase by increasing the loading non-proportionality.

Such drawbacks are overcome by using the RED criterion here proposed and presented in Section 3.2.

### 3.2. The Refined Equivalent Deformation (RED) Criterion

**Figure 7:**
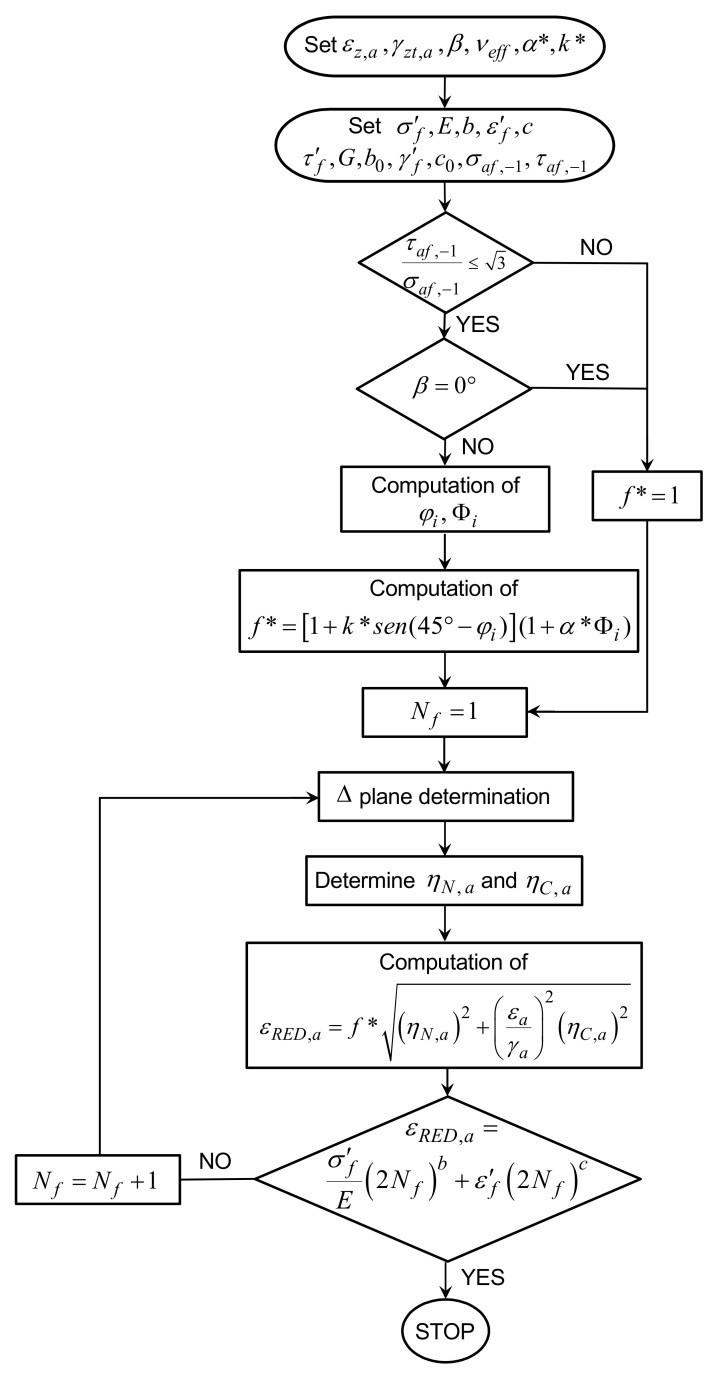
Flowchart of the RED criterion.

The flowchart of the RED criterion for biaxial fatigue test simulations is shown in Figure 7.

The RED criterion is a strain-based criterion, which is based on the critical plane concept and employs a novel enhancement factor in order to take into account the additional cyclic hardening under non-proportional fatigue loading. The main steps are detailed in the following.

Note that, in Figure 7, the loading non-proportionality is assumed to be produced by the phase shift (for simplicity of schematization), but the criterion is more general and it can be used in the presence of any cause of loading non-proportionality.

#### 3.2.1. Material Sensitivity to Loading Non-Proportionality

As previously mentioned, materials react in different ways to non-proportional loading under the same degree of non-proportionality. This can be described as the sensitivity of a given material to non-proportional loading.

Therefore, first of all, the criterion checks material sensitivity to non-proportional loading and the quotient of fatigue limits between fully reversed shear stress and fully reversed normal stress, $\tau_{af,-1}/\sigma_{af,-1}$, is used to achieve such an aim. As a matter of fact, according to Papadopoulos’ statement [1], when $\tau_{af,-1}/\sigma_{af,-1}\leq 1/\sqrt{3}$ the material shows a decreasing in fatigue limit under non-proportional loading.

Only in the case of sensitive materials, the criterion takes into consideration both the strain state and the additional cyclic hardening, by using a suitable strain factor, f*. Otherwise, such a factor is assumed to be equal to 1.0.

#### 3.2.2. Determination of the Critical Plane

The critical plane concept is based on the physical observation that cracks initiate and grow on specific material planes. There are different ways to define the critical plane itself. The orientation of the critical plane presented here is linked to both the directions of the principal strain axes and the mechanical/fatigue properties of the material, as detailed hereafter.

**Figure 8:**
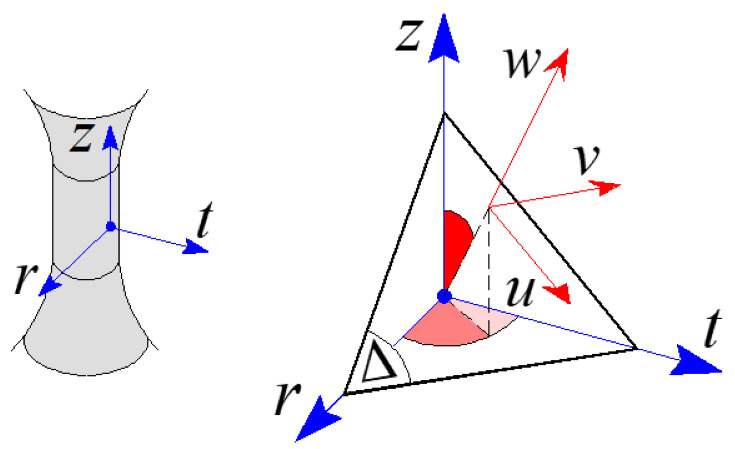
Global, rtz, and local, uvw frames.

Let us consider a material point of a smooth structural component under a fully reversed biaxial constant amplitude fatigue loading. The strain tensor at such a point is a function of:

(7) $$\varepsilon_{z}(t)=\varepsilon_{z,a}sen\left(2\pi \frac{t}{T}\right)$$

(8) $$\gamma_{zt}(t)=\gamma_{zt,a}sen\left(2\pi \frac{t}{T}-\beta \right)$$

with rtz being a fixed frame with its origin in the verification point, the r- and t-axes are perpendicular and tangential to the specimen surface, respectively, and the z-axis forms, with r and t, an orthogonal frame (Figure 8). The effective Poisson’s ratio needed to compute the whole strain tensor is here assumed to be equal to 0.5 [18].

According to [18], the critical plane, Δ, has to be determined first. Principal strains $\varepsilon_{1},\varepsilon_{2}$ and $\varepsilon_{3}$ are related to the 1,2 and 3 principal strain directions, respectively, and are computed during a loading cycle (with a period equal to T). The direction of the 1-axis in correspondence with the time instant, t, when $\varepsilon_{1}$ attains its maximum value is assumed as the normal to the fracture plane, $\hat{1}$. The normal w to the critical plane is assumed to form angle δ with respect to $\hat{1}$, and the rotation performed around the $\hat{2}$ direction is clockwise towards $\hat{3}$. Details on such an assumption are given in Reference [18].

The expression of the δ angle is as follows:

(9) $$\delta =\frac{3}{2}\left\{1-{\left[\frac{1}{2(1+\nu_{eff})}\frac{\gamma_{a}}{\varepsilon_{a}}\right]}^{\;2}\right\}45{}^{\circ}$$

where $\varepsilon_{a}$ and $\gamma_{a}$ (function of the number of loading cycles to failure, $N_{f}$) are computed by means of the tensile and torsional Manson–Coffin equations, that is:

(10) $$\varepsilon_{a}(N_{f})=\frac{{\sigma^{\prime }}_{f}}{E}{\left(2N_{f}\right)}^{b}+{\varepsilon^{\prime }}_{f}{\left(2N_{f}\right)}^{c}$$

(11) $$\gamma_{a}(N_{f})=\frac{{\tau^{\prime }}_{f}}{G}{\left(2N_{f}\right)}^{b_{0}}+{\gamma^{\prime }}_{f}{\left(2N_{f}\right)}^{c_{0}}$$

It can be observed that the definition of the orientation of the critical plane, given by Equation (9), is able to precisely capture the experimental fracture nature because it is a function of $N_{f}$. As a matter of fact:

(i) when the fracture is extremely ductile, that is $N_{f}\to 0$, the angle δ tends to be 45°;
(ii) when the fracture is extremely brittle, that is $N_{f}\to \infty$, the angle δ tends to be 0°;
(iii) between the above fracture types, 0°<δ<45°.

The critical plane is the plane where the fatigue assessment is performed, that is, where the damage parameter is computed.

#### 3.2.3. Damage Parameter under Proportional Loading

Let us consider a local frame uvw on the Δ plane (Figure 8), with its origin in the verification point. The u-axis is represented by the intersection of Δ and the wz plane, and the *v*-axis forms an orthogonal frame with *u* and *w*.

The displacement vector related to the verification point, η, may be decomposed in: a normal vector, $\eta_{N}$, function of the strain tensor component, $\varepsilon_{w}$, and a tangential vector, $\eta_{C}$, function of the strain tensor components, $\gamma_{wu}$ and $\gamma_{wv}$. For such vectors, their amplitudes are calculated, and more precisely: for $\eta_{N}$, its direction is fixed with respect to time and the amplitude $\eta_{N,a}$ is given by:

(12) $$\eta_{N,a}=\frac{1}{2}\;\;\left[\underset{0\leq t<T}{max}\left\|\eta_{N}(t)\right\|-\underset{0\leq t<T}{min}\left\|\eta_{N}(t)\right\|\right]$$

whereas the amplitude of $\eta_{C}$, $\eta_{C,a}$, is computed according to the maximum rectangular hull method proposed by Araujo et al. [44].

Note that in Reference [18] an analytical procedure to determine both $\eta_{N,a}$ and $\eta_{C,a}$ is presented, as an alternative to that employed here.

Under multiaxial proportional loading, the equivalent strain amplitude (damage parameter), $\varepsilon_{eq,a}$, is computed as follows:

(13) $$\varepsilon_{eq,a}=\sqrt{{\left(\eta_{N,a}\right)}^{2}+\frac{\varepsilon_{a}}{\gamma_{a}}{\left(\eta_{C,a}\right)}^{2}}$$

By implementing the tensile Manson-Coffin equation (see Equation (10)) in the fatigue limit condition represented by Equation (13), that is:

(14) $$\varepsilon_{eq,a}=\frac{{\sigma^{\prime }}_{f}}{E}{\left(2N_{f}\right)}^{b}+{\varepsilon^{\prime }}_{f}{\left(2N_{f}\right)}^{c}$$

the fatigue life, $N_{f}$, is determined.

#### 3.2.4. Damage Parameter under Non-Proportional Loading

The lifetime estimation performed by means of Equation (14) is relatively poor under non-proportional loading, due to the fact that the above equation is not able to take into account the additional cyclic hardening experimentally observed in materials sensitive to non-proportionality.

Therefore, a refined equivalent deformation amplitude, $\varepsilon_{RED,a}$, is here proposed by implementing in Equation (14) a novel strain factor, f*, and the fatigue limit condition is given by:

(15) $$\varepsilon_{RED,a}=f*\varepsilon_{eq,a}=\frac{{\sigma^{\prime }}_{f}}{E}{\left(2N_{f}\right)}^{b}+{\varepsilon^{\prime }}_{f}{\left(2N_{f}\right)}^{c}$$

where the factor, f*, is:

(16) $$f*=\left[1+k*sen\left|45{}^{\circ}-\varphi_{i}\right|\right](1+\alpha *\Phi_{i})$$

being $\varphi_{i}$ and $\Phi_{i}$ computed according to definitions given in Section 3.1. k* is given by:

(17) $$k*=\sum_{i=1}^{M}\frac{1}{sen\left|45{}^{\circ}-\varphi_{i}\right|}\left[\frac{2\varepsilon_{a}(N_{exp})}{\Delta \varepsilon_{ASME,i}}-1\right]$$

being *M* the test number under proportional loading by excluding those characterised by a strain path along 45°- direction, $\varepsilon_{a}(N_{exp})$ is computed by applying Equation (10), and $\Delta \varepsilon_{ASME,i}$ is calculated by means of Equation (2). Moreover, α* is given by:

(18) $$\alpha *=\sum_{i=1}^{N}\frac{1}{\Phi_{i}}\left[\frac{2\varepsilon_{a}(N_{exp})}{\Delta \varepsilon_{ASME,i}}-1\right]$$

where *N* is the tests number under non-proportional loading.

It can be observed that such equations are analogous to those proposed by Borodii et al. [3,4,5] (see Equations (4) and (5)); however, according to the author’s proposal, k* is computed by considering the angles formed between the uniaxial strain paths and the multiaxial proportional one (i.e., that with an orientation equal to 45°). As a matter of fact, such a parameter, according to the definition given in Section 3.1, should be representative of the difference in cyclic properties with respect to the proportional strain path; therefore, the reference orientation proposed here to compute k* is the multiaxial proportional one, contrary to that assumed by Borodii, and corresponding to the material principal direction [3,4,5].

This proposal allows to overcome one of the drawbacks presented in Section 3.1.2. Since both terms of Equation (16) increase by increasing the phase shift, the strain factor f* increases even by increasing β.

#### 3.2.5. Theoretical Results

First, for the material being examined, the sensitivity to non-proportional loading is checked, according to the theoretical statement by Papadopoulos [1]. The fatigue limits are derived from the elastic parameters of the both tensile and torsional Mason-Coffin curves [45]. More precisely, $\tau_{af,-1}$ is equal to 261.45 MPa, whereas $\sigma_{af,-1}$ is equal to 490.08 MPa, the above limits being referred to $10^{7}$ loading cycles (according to Reference [46]). Consequently, $\tau_{af,-1}/\sigma_{af,-1}=0.53$, that is the material being examined can be considered sensitive to non-proportional loading.

**Figure 9:**
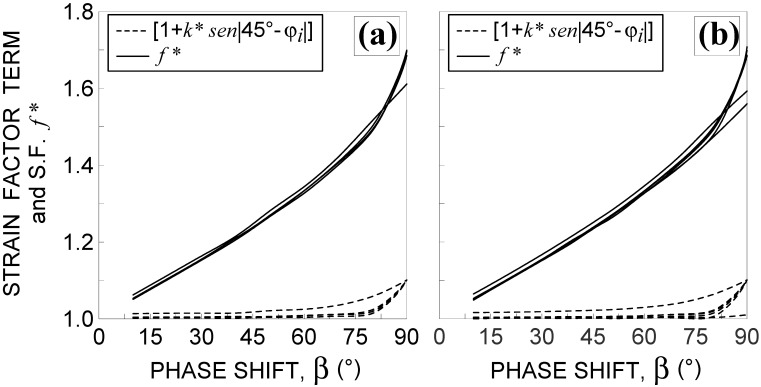
Influence of the phase shift $\beta \;\left(10{}^{\circ}\leq \beta \leq 90{}^{\circ}\right)$ on both the 1st term of the strain factor f* (see Equation (16)) and f* for the non-proportional loading conditions: (**a**) Nos. 7–12 and (**b**) Nos. 13–18, in Table 2.

**Figure 10:**
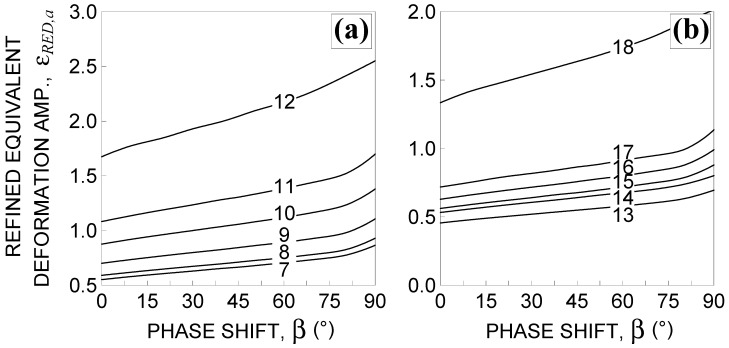
Influence of the phase shift $\beta \;\left(0{}^{\circ}\leq \beta \leq 90{}^{\circ}\right)$ on the refined equivalent deformation amplitude for the loading conditions: (**a**) Nos. 7–12 and (**b**) Nos. 13–18, in Table 2.

In Figure 9, the first term in the brackets of Equation (16) and strain factor f* are plotted against β for all examined non-proportional loading conditions. Figure 10 shows the corresponding trends of the refined equivalent deformation amplitude, $\varepsilon_{RED,a}$, including the proportional loading condition (that is β=0°).

**Figure 11:**
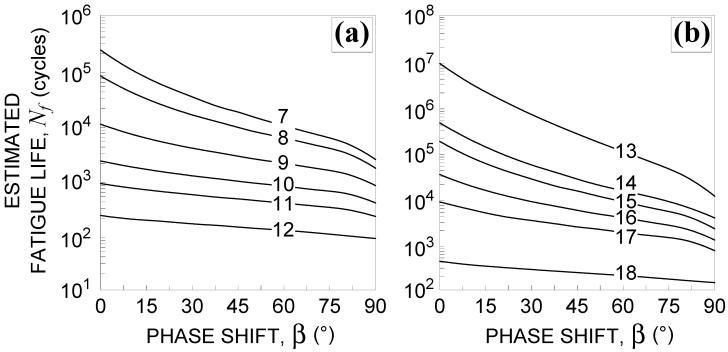
Influence of the phase shift $\beta \;\left(0{}^{\circ}\leq \beta \leq 90{}^{\circ}\right)$ on fatigue life estimation using the refined equivalent deformation criterion for the loading conditions: (**a**) Nos. 7–12 and (**b**) Nos. 13–18, in Table 2.

From Figure 9, it can be observed that one of the drawbacks of the reduced strain range method is overcome, since $\varepsilon_{RED,a}$ increases along with an increasing β (Figure 10). The lifetime, $N_{f}$, decreases up to a minimum in correspondence of 90°, as shown Figure 11, in accordance with the experimental evidence. Therefore, the second drawback is also overcome.

**Figure 12:**
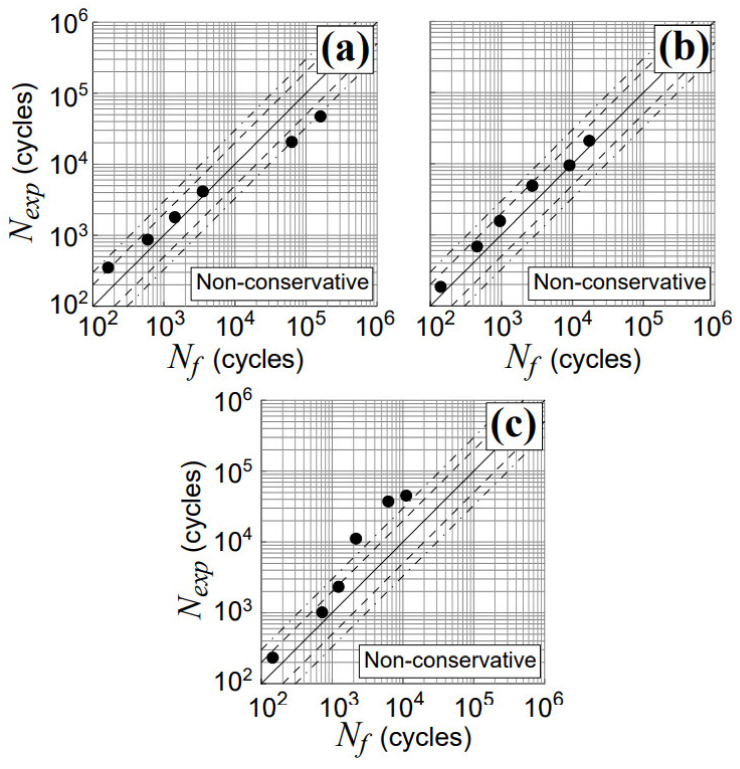
Experimental and estimated lifetime of TC4 titanium alloy obtained using the refined equivalent deformation criterion for tests: (**a**) Nos. T1–T6, (**b**) Nos. T7–T12, and (**c**) Nos. T13–T18, in Table 2.

For each multiaxial test listed in Table 2, the fatigue life is computed and compared with the experimental one in Figure 12. It can be observed that, under proportional loading (Figure 12a), 67% of the results fall within scatter band 2, whereas 83% of the results fall within scatter band 3. Under non-proportional loading and β=45° (Figure 12b), 100% of the results fall within scatter band 2, whereas for β=90° (Figure 12c), 50% of the results fall within scatter band 2 and the other 50% fall out of scatter band 3. Moreover, under non-proportional loading, conservative results are obtained.

The accuracy of the proposed criterion can also be evaluated by means of the mean square error [18], $T_{RMS}$. By considering all tests (that is, No. T1 to No. T18, in Table 2) the value is equal to 2.34. The accuracy is slightly greater than that characterizing the Borodii et al. method, being $T_{RMS}$ equal to 2.42.

It is worth mentioning that, according to the root mean square error method, the value of $T_{RMS}$ is equal to 1 when a perfect correspondence exists between experimental and estimated fatigue lifetimes. On the other hand, if all the computed results fall within scatter band 2, the value would be lower than 2, while if all the computed results fall within scatter band 3, the value would be lower than 3.

**Figure 13:**
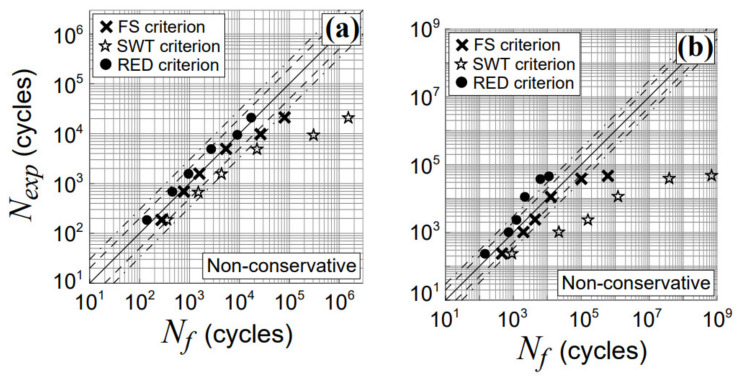
Experimental and estimated lifetimes obtained for TC4 titanium alloy using the FS criterion [47] and the SWT criterion [48] for non-proportional tests with a phase shift, β, equal to (**a**) 45° (Nos.T7–T12, in Table 2) and (**b**) 90° (Nos.T13–T18, in Table 2).

Finally, the above results are compared with those obtained by using two other criteria, available in the literature, the Fatemi and Socie (FS) criterion [47], and the SmithWatson and Topper (SWT) criterion [48], as shown in Figure 13. The RED criterion accuracy is greater with respect to that related to each of the above examined criteria.

## 4. Conclusions

In the present paper, a novel criterion, the RED criterion, has been proposed in order to estimate the fatigue lifetime of materials that are sensitive to non-proportionality.

Due to the fact that, in such conditions, a fatigue criterion cannot accurately estimate fatigue life by considering only the stress/strain state inside a material, an enhancement factor, implemented into the damage parameter relationship, has been employed. More precisely, along the line of the strain factor definition implemented in the reduced strain range method by Borodii et al.; the strain factor proposed here is a function of material constants, strain path orientation, and the degree of non-proportionality.

The use of such a strain factor allows to overcome the drawbacks of the Borodii method, and to define a refined equivalent deformation amplitude that represents the experimental data with a quite satisfactory accuracy, especially under non-proportional loading.

More precisely, the proposed criterion has been applied to experimental data obtained by testing TC4 titanium alloy specimens under multiaxial LCF. In such a context, the accuracy of the criterion has been computed by means of the mean square error, obtaining satisfactory results, being the value of $T_{RMS}$ equal to 2.34. The comparison with other criteria available in the literature has shown that the novel criterion holds a greater accuracy.

Further investigations on other metallic materials, sensitive to additional cyclic hardening, are in progress.

## Figures and Tables

**Table 1:** Chemical composition of TC4 titanium alloy (in wt %) [6].

Material	Al	V	Fe	C	N	H	O	Ti
TC4	6.4	4.1	0.2	0.01	0.01	0.002	0.16	Balance

**Table 2:** Experimental results under multiaxial loading [6]. The values of $\varphi_{i}$, $\Phi_{i}$, $\Delta \varepsilon_{ASME,i}^{}$, (i=1,…,18) for each strain path are reported.

TEST No.	$\varepsilon_{a}$ (%)	$\gamma_{a}$ (%)	β (°)	$N_{exp}$ (Cycles)	φ (°)	Φ (-)	$\Delta \varepsilon_{ASME}$ (%)	$N_{f}$ (Cycles)
**T1**	0.345	0.648	0	47,195	45	0	1.018	84,402
**T2**	0.427	0.710	0	20,611	45	0	1.184	43,305
**T3**	0.576	0.938	0	4141	45	0	1.581	12,059
**T4**	0.687	1.111	0	1795	45	0	1.880	5617
**T5**	0.863	1.371	0	868	45	0	2.342	2127
**T6**	1.391	2.038	0	351	45	0	3.644	302
**T7**	0.391	0.643	45	20,953	43.143	0.412	0.996	8434
**T8**	0.418	0.702	45	9478	43.744	0.414	1.076	5922
**T9**	0.496	0.831	45	4898	43.675	0.414	1.275	2800
**T10**	0.620	1.043	45	1563	43.792	0.414	1.597	1033
**T11**	0.772	1.255	45	683	42.813	0.411	1.957	431
**T12**	1.224	1.756	45	185	37.811	0.403	2.947	79
**T13**	0.349	0.639	90	45,138	90.000	0.946	0.738	5434
**T14**	0.418	0.704	90	37,273	0.000	0.972	0.836	10,102
**T15**	0.499	0.821	90	11,152	0.000	0.950	0.998	4859
**T16**	0.556	0.934	90	2332	0.000	0.970	1.112	2881
**T17**	0.632	1.079	90	1017	0.000	0.986	1.264	1579
**T18**	1.229	1.700	90	233	0.000	0.799	2.458	129

## Data Availability

Data sharing not applicable.
